# Clinical outcomes and predictive factors of stent grafts treatment for symptomatic central venous obstruction in end stage kidney disease patients with arteriovenous access

**DOI:** 10.1038/s41598-024-63287-2

**Published:** 2024-06-03

**Authors:** Yamin Liu, Yufei Wang, Xinfang Wang, Beihao Zhang, Xiaoqing Lu, Xianhui Liang, Pei Wang

**Affiliations:** Blood Purification Center, Institute of Nephrology, the First Affiliated Hospital of Zhengzhou University, 1 East Jianshe Road, Zhengzhou, Henan Province 450052 People’s Republic of China

**Keywords:** Nephrology, Risk factors

## Abstract

To assess the efficacy of stent grafts (SGs) in managing central venous obstruction disease (CVOD) in hemodialysis (HD) patients with arteriovenous (AV) access, and to identify predictive factors influencing the SG treatment outcomes. HD subjects with CVOD who underwent SGs placement at our center between August 2018 and June 2022 were enrolled. Survival curve analysis using the Kaplan–Meier method and log-rank test was performed. Cox proportional hazards regression analysis was employed to identify predictive factors associated with outcomes. A total of 59 SG implantation procedures for CVOD were analyzed, comprising 30 cases of stenosis and 29 cases of occlusion. The access circuit primary patency (ACPP) at 6, 12, and 24 months post-SG placement were 80.9%, 53.8%, and 31.4%, respectively, while, the target lesion primary patency (TLPP) were 91.3%, 67.6%, and 44.5%, respectively. Subgroup analysis revealed higher TLPP in the stenosis group compared to the occlusion group, although the difference was not statistically significant (*P* = 0.165). The TLPP was significantly improved by SG placement in those who had antecedent balloon dilations (*P* < 0.001). Cox proportional hazards regression identified target lesion length ≥ 30 mm and procedure defects as independent predictors of lower TLPP after SG treatment for CVOD in HD patients. SG placement demonstrates safety and efficacy in managing CVOD among HD patients, leading to improved TLPP of endovascular therapy (EVT) for CVOD. Notably, long target lesions (≥ 30 mm) and procedure defects emerged as predictive factors influencing TLPP.

## Introduction

Central venous obstruction disease (CVOD) represents a pathological condition in which stenosis or occlusion occurs in the central vein system, primarily involving the superior vena cava (SVC), right and left brachiocephalic veins (BCV) and subclavian veins (SCV). The prevalence of CVOD ranges from 5% to as high as 50% in patients with end-stage kidney disease (ESKD) undergoing hemodialysis (HD)^1–3^. The predominant cause of CVOD is the insertion of central venous catheter (CVC)^2^. However, an increasing incidence of CVOD is being observed due to the utilization of other endovascular devices such as pacemaker leads^4^. Typically, CVOD remains asymptomatic until the establishment of a functional arteriovenous fistula (AVF) or arteriovenous graft (AVG), leading to a significant increase in cardiac output and venous return^5,6^. Symptomatic presentation of CVOD may include ipsilateral upper extremity or breast edema, venous hypertension, varicosities, prolonged bleeding from access sites, or even the abandonment of vascular access^7^. These clinical manifestations underscore the importance of management of CVOD to prevent associated complications and optimize the efficacy of HD therapy.

Endovascular therapy (EVT) is recommended as the primary treatment for managing symptomatic CVOD^7^. Recent studies have demonstrated that stent grafts (SGs) have superior long-term patency of CVOD over percutaneous transluminal angioplasty (PTA) or bare stents to treat CVOD^8,9^. Whereas, considerable differences exist in the previous studies about the rates of target lesion primary patency (TLPP) following SG placement for CVOD, necessitating an in-depth investigation for associated factors influencing TLPP outcomes. Despite the growing recognition of the efficacy of SGs, there remains a paucity of studies investigating predictive factors influencing primary patency following SG placement in ESKD patients with CVOD^10,11^. In this study, we retrospectively reviewed the data of ESKD patients with symptomatic CVOD who underwent SG placement at our institution and accessed the predictive factors associated with TLPP outcomes.

## Method

### Study design and subjects screening

**Figure 1:**
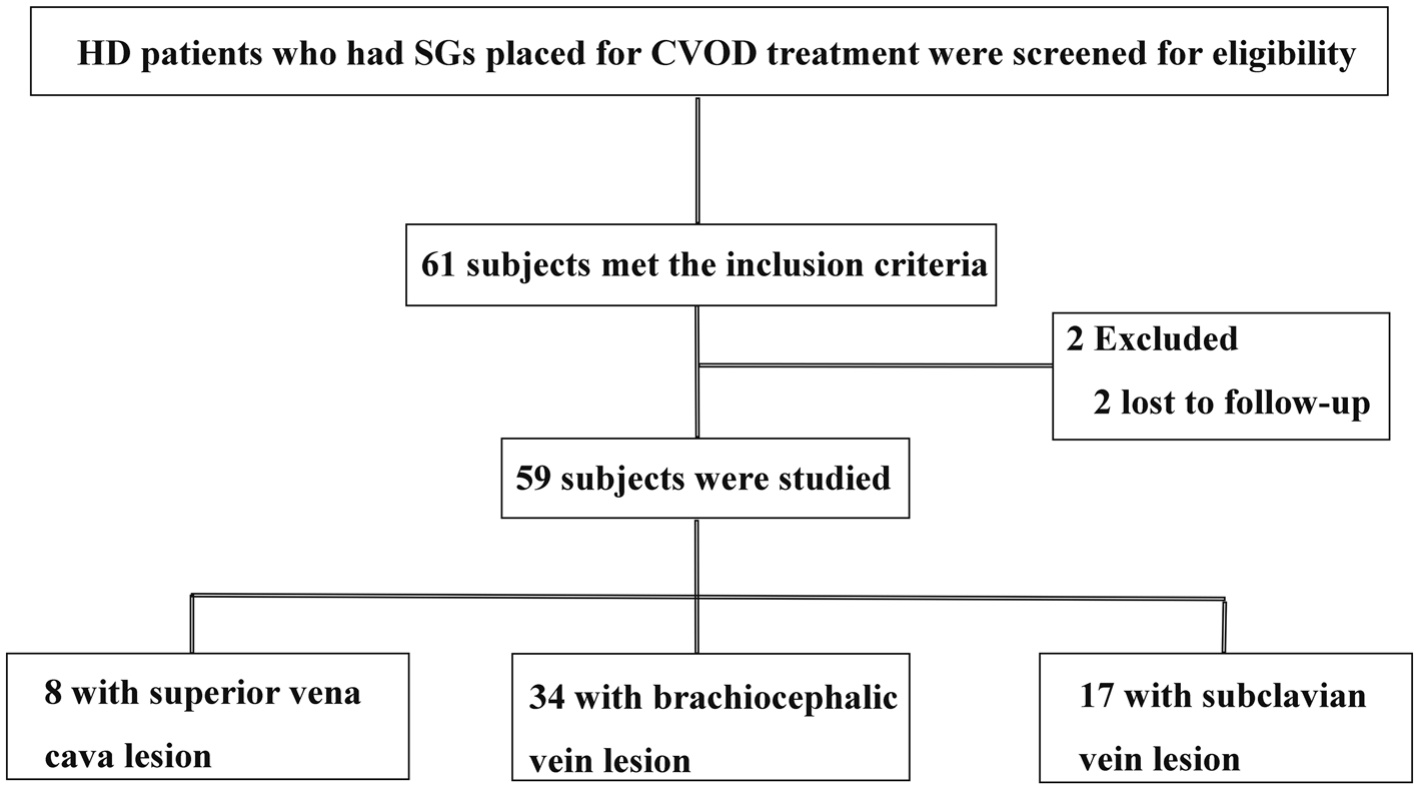
Flow diagram depicting the design of the retrospective cohort study.

This study was a retrospective study in a cohort who received SGs for CVOD treatment between August 2018 and June 2022 at the First Affiliated Hospital of Zhengzhou University, Zhengzhou, China. Subjects lost to follow-up were excluded (Fig. 1). CVOD was defined as symptomatic vascular stenosis or obstruction by lesions located in the central venous system comprising SCVs, BCVs, and SVCs. The indications for SG placement include occlusive lesions, acute elastic recoil > 30% after PTA, and stenosis relapsed within 3 months after PTA. Fifty-nine SG placement procedures were performed in 54 patients and 61 SGs were implanted in the cohort. Follow-up ended on December 31, 2022, access abandonment, patient death, or loss to follow-up. This study had been performed in accordance with the Declaration of Helsinki and was approved by the Human Research Ethics Committee of the First Affiliated Hospital of Zhengzhou University (Ethics approval number: 2022-KY-1497-002).

### Procedures

Informed consent was obtained from all patients prior to the procedure. All procedures were performed under local anesthesia. Digital subtraction angiography (DSA) was conducted before all EVT procedures to evaluate the lesions, and some were combined with computed tomography angiography (CTA). Viabahn™ (W. L. Gore & Associates Inc., CA, USA), Fluency ™ Plus (Bard Peripheral Vascular, Tempe, Arizona, USA), or Excluder™ AAA contralateral leg (W. L. Gore & Associates Inc., CA, USA) SGs, with a diameter matching the adjacent vessel and length covering the entire target lesion, were selected based on the physician’s judgement. The treatment was performed at a single center by a well-trained interventional nephrologist. The first choice of approach is AVF outflow or the venous limb of the AVG. However, the femoral vein or other approaches are needed in some cases. Unfractionated heparin (1000–5000 IU) was injected into the sheath to prevent thrombosis. We often used 0.035-inch guidewire cooperated with a single curved catheter or a long sheath to cross the severe stenosis lesion, known as the blunt recanalization technique. For the total occlusive lesion, we used a sharp recanalization technique^12^. SGs was performed after angioplasty using a noncompliant balloon. Procedure duration was defined as the interval between the onset of percutaneous puncture and the removal of vascular sheaths. Procedures were judged by two other operators to evaluate poor runoff or incomplete coverage of the lesion.

### Follow-up

All patients underwent regular HD. On-site or telephone surveillance was performed quarterly. Symptoms related to superior vena cava syndrome were the key points of routine surveillance, and venography were recommended when subjects were suspected to have significant restenosis. EVT was performed according to the indications recommended by the KDOQI Vascular Access guidelines^7^.

### Definition

Clinical success was defined as the ability to complete at least one HD session after SG placement. Patency was defined according to the Committee on Reporting Standards for Arteriovenous Access by the Society for Vascular Surgery and the American Association for Vascular Surgery. TLPP was defined as the interval from any EVT until reintervention in the target lesions. Access Circuit Primary Patency (ACPP) after SG refers to the interval from SG placement until reintervention in the access circuit^13,14^. Major complications were defined as those that required additional treatment.

### Statistical analysis

SPSS 23.0 and Prism 9.0 were applied for statistical processing. The measurement data were expressed as mean ± standard deviation or median (interquartile range), and the count data were expressed as the number of cases and percentages. Normality of the data was tested using the Kolmogorov–Smirnov method. For comparisons between two groups of measurement data, t-test or Mann–Whitney U tests was used to analysis. Comparison of count data was conducted using the χ^2^ test or Fisher’s exact test. The Kaplan–Meier method and log-rank test were used for survival curve analysis. Cox proportional hazards regression was used for predictive factor analysis of SG patency. Statistical significance was set at *P* < 0.05.

## Results

### Baseline characteristics of patients, lesions and procedures

In this cohort, 54 HD patients underwent 59 SG implantation procedures for CVOD. The baseline characteristics of subjects lesions and procedures are presented in Table 1. The mean age of the subjects was 59.3 ± 1.6 years. 36 (61.0%) subjects were male, and 43 (72.9%) had diabetes mellitus. 35 (59.3%) subjects had a history of ipsilateral CVC insertion, and the interval of catheter indwelling was 2 (0.4, 10) months. Most subjects (47 cases, 79.7%) had AVFs and other 12 subjects (20.3%) had AVGs (Supplementary Table S1). Arm swelling was the main manifestation of CVOD. Most lesions (34 lesions, 57.6%) were in the BCVs and eight (13.6%) ones were in the SVCs. The interval between access creation and SG placement was 32.5 (17.7, 51.9) months. 38 (64.4%) subjects underwent at least one PTA intervention prior to SG placement. Indications for SG placement were total occlusion (49.2%) or stenosis (50.8%). The outflow of the AVF or venous limb of the AVG was the approach for most procedures; Nevertheless, the femoral vein or other approaches were needed in 10 procedures.

**Table 1:** The baseline characteristics of subjects and lesions.

Variable	Values
Male, case (%)	36 (61.0)
Age, year	59.3 ± 1.6
Diabetes mellitus, case (%)	43 (72.9)
AV access type, case (%)	
AVF	47 (79.7)
AVG	12 (20.3)
Location of AV access, case (%)	
Left upper extremity	38 (64.4)
Right upper extremity	21 (35.6)
History of ipsilateral CVC insertion, case (%)	35 (59.3)
Interval of ipsilateral CVC indwelling, months	2 (0.4, 10)
Length of target lesion, mm	30 (20, 60)
Symptoms, case (%)	
Swelling of head, face and neck	12 (20.3)
Swelling of arm	45 (76.3)
Swelling of breast	2 (3.4)
High venous pressure	19 (32.2)
Location of target lesion, case (%)	
Superior vena cava	8 (13.6)
Brachiocephalic vein	34 (57.6)
Subclavian vein	17 (28.8)
Across costoclavicular joint, case (%)	
Yes	34 (57.6)
No	25 (42.4)
Defects of procedure, case (%)	
No	46 (78.0)
Yes	13 (22.0 )
Poor distal runoff	9
Incomplete coverage of target lesion	6
Branch covered by SG, case (%)	
No	15 (25.4)
Internal jugular vein	35 (59.3)
Contralateral brachiocephalic vein	4 (6.8)
Others	5 (8.5)
Target lesion, case (%)	
Stenosis	30 (50.8)
Occlusion	29 (49.2)
Blood flow pre-SG, ml/min	925 (780, 1200)
Blood flow post-SG, ml/min	1000 (870, 1230)
Types of SG	
Viabahn™	43 (72.9)
Excluder™	15 (25.4)
Fluency™ Plus	1 (1.7)
Diameter of SG, mm	13 (11, 13)
Length of SG, mm	70 (50, 100)
Pre-dilated balloon diameter, mm	12 (10, 14)
Post-dilated balloon diameter, mm	12 (10, 14)
Stent diameter/adjacent distal vessel diameter ratio	
< 1	14 (23.7)
= 1	12 (20.3)
> 1	33 (55.9)
Approaches	
Fistula only	49 (83.1)
Fistula + femoral vein	7 (11.9)
Fistula + femoral vein + jugular vein	2 (3.4)
Internal jugular vein only	1 (1.7)
Multiple approach imaging for stent positioning	
Yes	35 (59.3)
No	24 (40.7)
Procedure duration, minute	55 (40,70)
Times of endovascular treatment prior SG	
0	21 (35.6)
1	19 (32.2)
2	6 (10.2)
≥ 3	13 (22.0)
Sharp recanalization	
Yes	6 (10.2)
No	53 (89.8)

*AV* arteriovenous, *AVF* arteriovenous fistula, *AVG* arteriovenous graft, *CVC* central venous catheter, *SG* stent graft.

SGs were selected to match the diameters of adjacent healthy veins and cover the entire target lesion (Supplementary Table S2). Procedural success was achieved in all subjects, with 2 (3.4%) major complications. One subject had severely poor distal runoff and incomplete coverage of the target lesion after the first SG implantation and underwent a second SG during the procedure. Another subject with SVC occlusion underwent sharp recanalization, but the guidewire entered the extravascular space and re-entered the true lumen during sharp recanalization^15^, and two SGs were needed to cover all the target lesions. Stent migration and vascular rupture were not observed in this study. The clinical success rate was 100%, and symptoms relieved in all the patients. Nine SGs were judged as poor distal runoff and six ones were judged as incomplete coverage of target lesions; eventually, 13 SGs had defects in procedure.

### Patency after SGs placement

**Figure 2:**
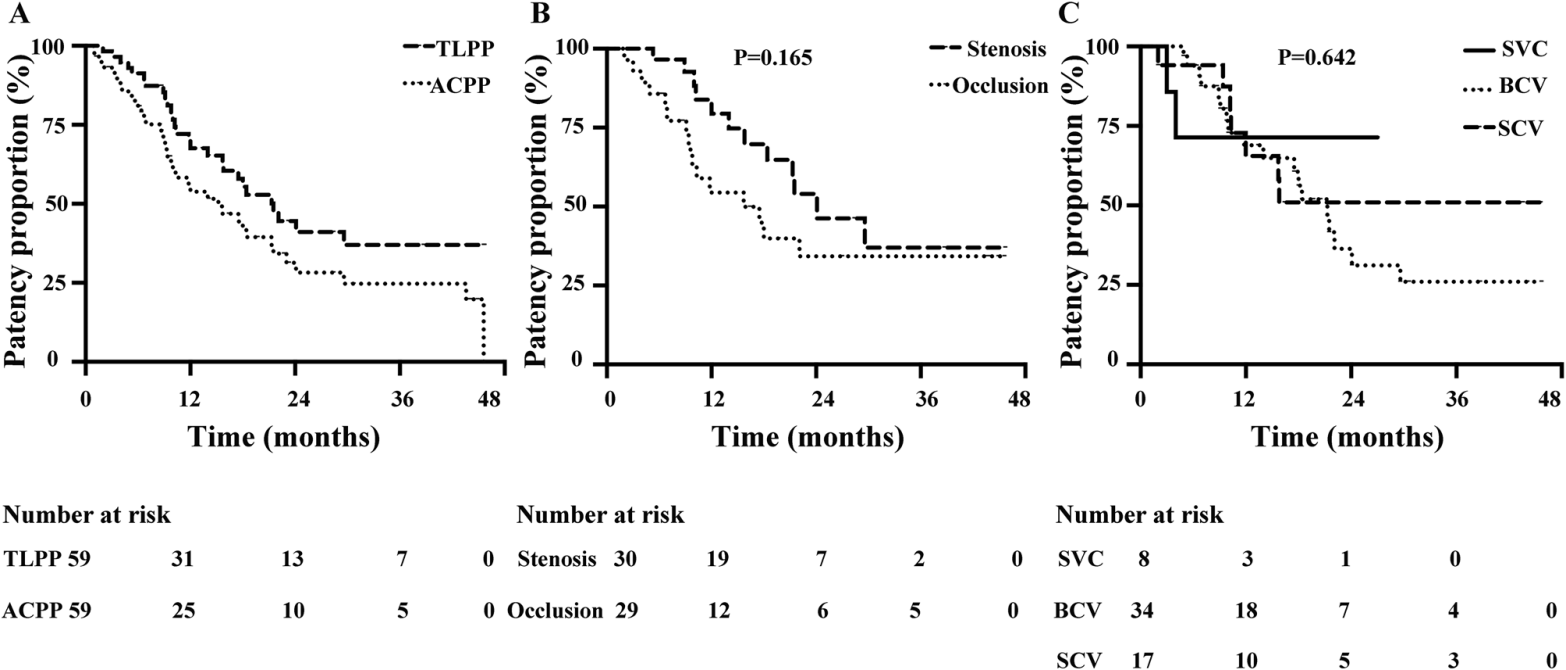
Kaplan–Meier curve of stent grafts (SGs). (**A**) Kaplan–Meier curves of target lesion primary patency (TLPP), access circuit primary patency (ACPP). (**B**) TLPP (stenosis VS. occlusion, *P* = 0.165). (**C**) TLPP (superior vena cava VS. brachiocephalic vein VS. subclavian vein, *P* = 0.642).

The subjects were follow-up to 23.6 ± 1.8 months. Six subjects died and other 2 subjects were lost to follow-up during follow-up. ACPP after SG placement at 6, 12, and 24 months were 80.9%, 53.8%, and 31.4%, respectively. The median of ACPP after SG placements was 15.2 (95% CI 8.3–22.1) months. TLPP after SG placement at 6, 12, and 24 months were 91.3%, 67.6%, and 44.5%, respectively. The median of TLPP after SG placements was 21.5 (95% CI 15.8–27.2) months (Fig. 2A and Table 2).

**Table 2:** Kaplan–Meier curve of stent grafts (SGs).

	6 months	12 months	24 months	36 months
TLPP (%)	91.3	67.6	44.5	37.0
ACPP (%)	80.9	53.8	31.4	24.7

*ACPP* access circuit primary patency, *TLPP* target lesion primary patency.

TLPP was higher in the stenosis group (n = 30) than in the occlusion group (n = 29), but the difference was not significant (*P* = 0.165) (Fig. 2B). TLPP of SGs in different locations was compared, and there was no significant difference among the three groups, i.e. SCV, BCV, and SVC (*P* = 0.642) (Fig. 2C).

### TLPP was improved by SGs

**Figure 3:**
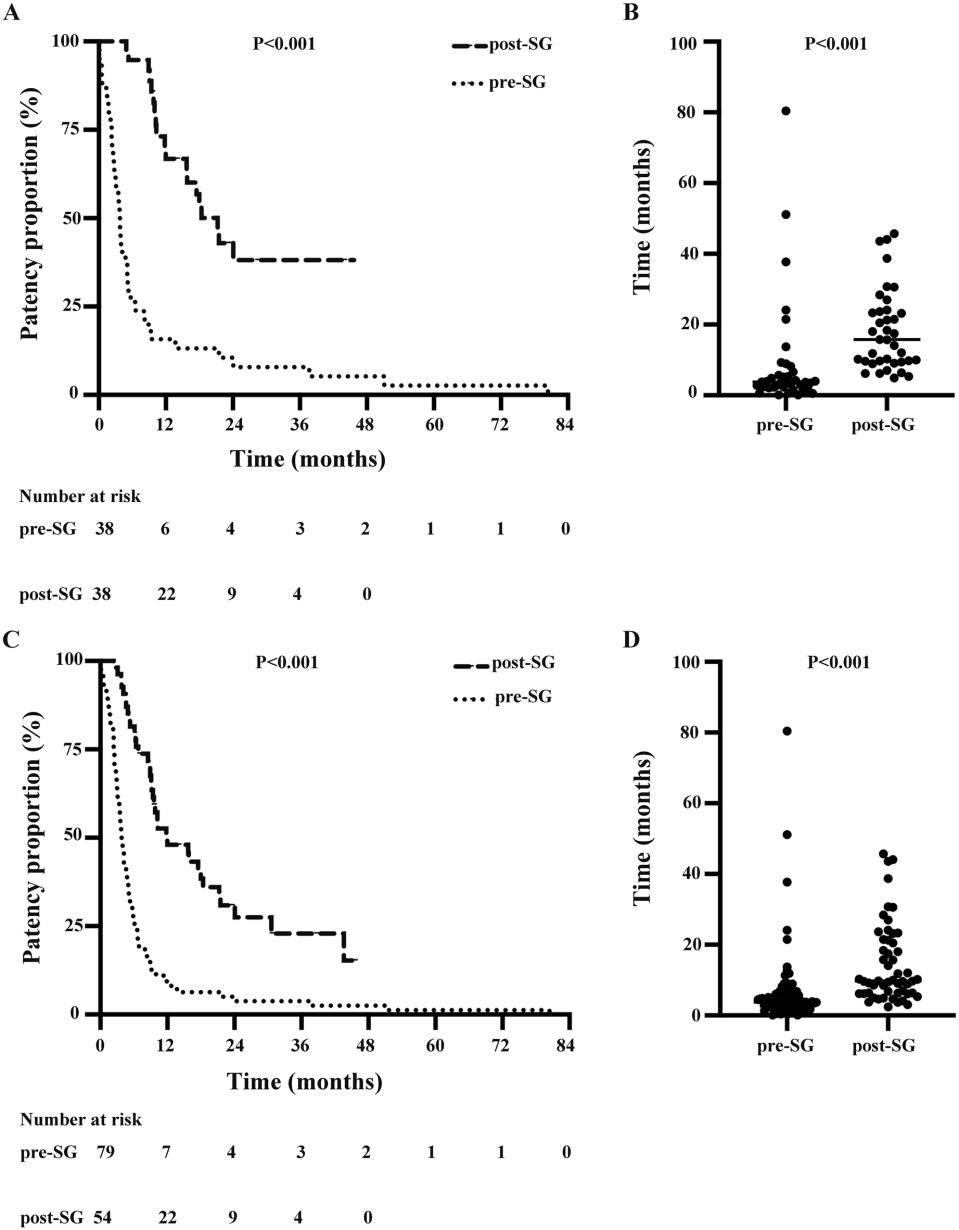
Improvement of target lesion primary patency (TLPP) post-SG placement. Among the 38 patients who had at least one percutaneous transluminal angioplasty (PTA) intervention prior SG placement, TLPP was significantly improved. (**A**) TLPP post-SG placement compared to proximate PTA pre-SG (*P* < 0.001); (**B**) Median intervention time post-SG is longer than the proximate PTA pre-SG [15.8 (9.6, 23.8) VS. 3.8 (2.3, 7) months, *P* < 0.001]; (**C**) TLPP was significantly improved after each intervention post-SG compared with pre-SG (*P* < 0.001), (**D**) Median of each intervention time were 3.8 (2.4, 6.3) months of pre-SG and 9.8 (6.3, 21.4) months of post-SG (*P* < 0.001).

TLPP of procedures before and after SG implantation was compared in a subgroup of 38 subjects who had at least one PTA prior to SG placement. TLPP of procedures post-SG implantation was higher than pre-SG implantation [9.8 (6.3, 21.4) vs. 3.8 (2.4, 6.3) months, *P* < 0.001] (Fig. 3).

### Predictive outcome factors of SGs

Cox proportional hazards analysis was used to investigate the impact of preoperative and perioperative factors on TLPP. Multivariate analysis revealed that target lesion length ≥ 30 mm and defects in the procedure were independent predictors of target lesion failure (Table 3).

**Table 3:** Univariate and multivariate Cox proportional hazards regression analyses of TLPP.

Variable	Univariate model		Multivariate model^a^		Multivariate model^b^		Multivariate model^c^	
	HR (95%CI)	P	HR (95%CI)	P	HR (95%CI)	P	HR (95%CI)	P
Sex (Female/Male)	0.880 (0.406–1.906)	0.745						
Age (≥ 57/ < 57 years)	0.726 (0.340–1.552)	0.409						
Diabetes mellitus (Yes/No)	0.842 (0.368–1.930)	0.685						
AV access type (AVG/AVF)	2.850 (1.206–6.736)	0.017	1.842 (0.632–5.371)	0.263	1.846 (0.601–5.666)	0.284	1.310 (0.394–4.362)	0.659
Location of AV access (Right/Left upper extremity)	1.366 (0.612–3.052)	0.446						
History of ipsilateral CVC insertion (Yes/No)	1.206 (0.552–2.638)	0.638						
Target lesion length (≥ 30/ < 30 mm)	3.741 (1.663–8.412)	0.001	4.558 (1.545–13.448)	0.006	4.578 (1.268–16.526)	0.020	5.914 (1.525–22.937)	0.010
Location of target lesion		0.645						
Superior vena cava	1							
Brachiocephalic vein	1.604 (0.370–6.960)	0.528						
Subclavian vein	1.129 (0.233–5.475)	0.880						
Across costoclavicular joint (Yes/No)	0.327(0.151–0.706)	0.004	0.615 (0.220–1.724)	0.355	0.616 (0.214–1.776)	0.370	0.438 (0.135–1.417)	0.168
Defects of procedure (Yes/No)	11.822 (4.860–28.757)	< 0.001	14.020 (3.687–53.317)	< 0.001	14.022 (3.678–53.324)	< 0.001	13.960 (3.571–54.573)	< 0.001
Stent diameter/adjacent distal vessel diameter ratio (≥ 1/ < 1)	4.036 (0.954–17.075)	0.058	2.350 (0.511–10.814)	0.273	2.349 (0.510–10.815)	0.273	2.175 (0.458–10.320)	0.328
Length of SG (≥ 100/ < 100 mm)	1.733 (0.802–3.745)	0.162			0.993 (0.307–3.208)	0.990	1.082 (0.322–3.635)	0.899
Branch covered by SG (Yes/No)	0.929 (0.374–2.307)	0.873						
Multiple approach imaging for stent positioning (Yes/No)	1.430 (0.640–3.193)	0.383						
Target lesion type (Occlusion/Stenosis)	1.704 (0.796–3.648)	0.170	1.769 (0.760–4.120)	0.186	1.769 (0.758–4.127)	0.187	1.295 (0.499–3.361)	0.595
Blood flow post-SG (≥ 1000/ < 1000 ml/min)	0.790 (0.365–1.709)	0.549						
Approach (Other routes/Fistula only)	1.566 (0.652–3.763)	0.316						
EVT prior SG (Yes/No)	0.936 (0.409–2.142)	0.875						
Sharp canalization (Yes/No)	1.687 (0.637–4.468)	0.292					3.382 (0.900–12.704)	0.071

*AV* arteriovenous, *AVF* arteriovenous fistula, *AVG* arteriovenous graft, *CI* confidence interval, *CVC* central venous catheter, *EVT* endovascular treatment, *HR* hazard ratio, *SG* stent graft, *TLPP* target lesion primary patency; The cut-off of age was median value. Model^a^, Variables with a value of *p* < 0.1 in the univariate analysis were input into the multivariate Cox regression analysis.

Model^b^, Variables with a value of *p* < 0.2 in the univariate analysis were input into the multivariate Cox regression analysis.

Model^c^, Variables with a value of *p* < 0.3 in the univariate analysis were input into the multivariate Cox regression analysis.

## Discussion

CVOD represents a crucial concern for HD patients^16^. The escalating prevalence of CVOD can be attributed primarily to the persistent utilization of CVCs as the primary access modality for HD^2,17^. Managing CVOD poses significant challenges with occasionally necessitating access abandonment. EVT emerged as the preferred approach for CVOD management due to the limitations for surgical interventions, notably the thoracic location of lesions, the advanced age and high comorbidity burden frequently observed in HD patients^18–21^. However, EVT is not with its drawbacks, including issues such as elastic recoil and restenosis following PTA, which can markedly affect the quality of life and increase medical expense^22–24^. Studies have demonstrated the superiority of SGs over both PTA and bare-metal stents in terms of long-term patency^9,23,25^.

In this study, we analyzed the patency of 59 SG implantation procedures in CVOD. This study demonstrated that the ACPP at 6, 12, and 24 months post-SG placement were 80.9%, 53.8%, and 31.4%, respectively. The TLPP at corresponding time points were 91.3%, 67.6%, and 44.5%, respectively. A number of previous studies provide valuable insights into the efficacy of SGs in CVOD management. For instance, a cohort study including 60 Excluder SGs in CVOD reported an ACPP of 54.9% and TLPP of 88.3% at 12 months^26^. Additionally, another study showed that TLPP and ACPP of 56% and 29%, respectively, at 12 months post-SG placement in CVOD^9^. Moreover, a study of 30 Viabahn SGs in CVOD reported TLPP of 97%, 81%, 67%, and 45% at 3, 6, 12, and 24 months, respectively^8^. Collectively, these findings underscore the favorable long-term patency outcomes associated with SG utilization in CVOD management.

In subgroup analysis, although the difference did not achieve statistical significance, the TLPP of SGs in the stenosis group exhibited superiority over that in the occlusive group. This suggests that the importance of early identification and treatment of lesions at the stenotic stage, potentially averting progression to complete occlusion. Consistently, Jones et al.^8^ reported that patients with occlusive lesions had a significantly shorter primary patency interval compared to those with stenosis (*P* = 0.05). Occluded veins were more likely to require SGs (*P* = 0.02). Additionally, procedural duration was notably prolonged in the occlusive group compared to the stenosis group [65(45,107.5) vs. 45(38,60) min, *P* = 0.002]. Our study also revealed that SG placement not only enhanced the intervention interval but also reduced the frequency of interventions in patients who had previously undergone PTA. In line with the investigation of Jones et al., SG placement for CVOD in HD patients proved safe and effective following failed PTA attempts^8^. Moreover, Gong et al. concluded that SG placement effectively salvaged central vein stenosis with recalcitrant restenosis in patients with AVF when PTA failed^27^. Our results were consistent with Vachharajani et al.’s assertion that SG implantation confers benefits to TLPP and has been shown to reduce the frequency of interventions to maintain access patency^28^.

Our study revealed that a target lesion length ≥ 30 mm, rather than stent length, was a predictive factor for poor outcome. Previous studies have suggested stent length as a predictive factor^11^, while our study findings did not corroborate this association. In our study, six SGs did not completely cover all the culprit lesions due to the accessibility of the stents. The selection of stent length was depended on several factors, including the length of the culprit lesions, the presence of adequate runoff post-release, and coverage of critical branches. So, it is reasonable to speculate that the length of culprit lesion rather than the stent length, served as the predictive factor for poor outcome. Our results also identified procedural defects as another predictive factor. For optimal distal runoff grafts, SGs and the vessel are consistent without serious angulation, which minimizing blood flow disturbances at the stent edge, thus reduce the probability of distal edge stenosis of the SGs. Another predictive factor was the incomplete coverage of lesions, which means intimal hyperplasia of the culprit lesion will continue to cause stenosis, potentially aggravated by the disordered blood flow at the stent edge^9^.

Previous study reported the undersized stents were superior to the same-sized or oversized stents for central venous lesions^29^. However, our findings did not align with this conclusion. Precise measurement of the diameter of central veins was difficult for the features of large lumen, thin wall, prone to be influenced by breathing and big difference between segments. At times, the diameter of stent may appear oversized relative to the distal margin of lesion but undersized relative to the proximal margin. The precise selection of stents depended on the advancements in measurement techniques and invocation of variable dimension stents.

The present study is not without limitations. Firstly, it was a retrospective study conducted at a single center, which would may compromise the reproducibility of the findings. Secondly, the relatively short follow-up duration in our study may restrict the comprehensive assessment of long-term outcomes. Therefore, prospective randomized controlled trials are imperative to provide further insight into the effectiveness and safety of SG treatment for CVOD.

## Conclusion

In conclusion, SG placement in CVOD of HD patients is safe and effective and could improve access patency. Long target lesion (≥ 30 mm) and procedure defects are predictors of low TLPP.

### Supplementary Information

Supplementary Tables.

## Data Availability

The datasets used and/or analyzed during the current study available from the corresponding author on reasonable request.

## References

[1] Beathard GA (1992). Percutaneous transvenous angioplasty in the treatment of vascular access stenosis. Kidney Int..

[2] MacRae JM, Ahmed A, Johnson N, Levin A, Kiaii M (2005). Central vein stenosis: A common problem in patients on hemodialysis. ASAIO J..

[3] Agarwal AK (2013). Central vein stenosis. Am. J. Kidney Dis..

[4] Chuang CL, Tarng DC, Yang WC, Huang TP (2001). An occult cause of arteriovenous access failure: Central vein stenosis from permanent pacemaker wire. Report of three cases and review of the literature. Am. J. Nephrol..

[5] Oguzkurt L, Tercan F, Yildirim S, Torun D (2005). Central venous stenosis in haemodialysis patients without a previous history of catheter placement. Eur. J. Radiol..

[6] Morosetti M (2000). Late symptomatic venous stenosis in three hemodialysis patients without previous central venous catheters. Artif. Organs.

[7] Lok CE (2020). KDOQI clinical practice guideline for vascular access: 2019 Update. Am. J. Kidney Dis..

[8] Jones RG, Willis AP, Jones C, McCafferty IJ, Riley PL (2011). Long-term results of stent-graft placement to treat central venous stenosis and occlusion in hemodialysis patients with arteriovenous fistulas. J. Vasc. Interv. Radiol..

[9] Anaya-Ayala JE (2011). Efficacy of covered stent placement for central venous occlusive disease in hemodialysis patients. J. Vasc. Surg..

[10] Chen B (2022). One-year outcomes and predictive factors for primary patency after stent placement for treatment of central venous occlusive disease in hemodialysis patients. Ther. Adv. Chron. Dis..

[11] Boutrous ML (2019). Stent-graft length is associated with decreased patency in treatment of central venous stenosis in hemodialysis patients. Ann. Vasc. Surg..

[12] McDevitt JL (2019). Approach, technical success, complications, and stent patency of sharp recanalization for the treatment of chronic venous occlusive disease: Experience in 123 patients. Cardiovasc. Interv. Radiol..

[13] Schmidli J (2018). Editor's choice—vascular access: 2018 Clinical practice guidelines of the european society for vascular surgery (ESVS). Eur. J. Vasc. Endovasc. Surg..

[14] Sidawy AN (2002). Recommended standards for reports dealing with arteriovenous hemodialysis accesses. J. Vasc. Surg..

[15] Wang Y, Liang X, Zhou CY, Lu X, Wang P (2022). Two cases of guidewire entering extravascular space then reentering the true lumen during sharp recanalization of superior vena cava occlusion. Chin. J. Nephrol..

[16] Agarwal AK, Khabiri H, Haddad NJ (2017). Complications of vascular access: Superior vena cava syndrome. Am. J. Kidney Dis..

[17] Barrett N, Spencer S, McIvor J, Brown EA (1988). Subclavian stenosis: A major complication of subclavian dialysis catheters. Nephrol. Dial. Transplant..

[18] Sfyroeras GS (2017). A review of open and endovascular treatment of superior vena cava syndrome of benign aetiology. Eur. J. Vasc. Endovasc. Surg..

[19] Kalra M (2003). Open surgical and endovascular treatment of superior vena cava syndrome caused by nonmalignant disease. J. Vasc. Surg..

[20] Rizvi AZ (2008). Benign superior vena cava syndrome: Stenting is now the first line of treatment. J. Vasc. Surg..

[21] Auyang PL, Chauhan Y, Loh TM, Bennett ME, Peden EK (2019). Medial claviculectomy for the treatment of recalcitrant central venous stenosis of hemodialysis patients. J. Vasc. Surg. Venous Lymphat. Disord..

[22] Yildiz I (2019). The efficacy of paclitaxel drug-eluting balloon angioplasty versus standard balloon angioplasty in stenosis of native hemodialysis arteriovenous fistulas: An analysis of clinical success, primary patency and risk factors for recurrent dysfunction. Cardiovasc. Interv. Radiol..

[23] Haskal ZJ (2010). Stent graft versus balloon angioplasty for failing dialysis-access grafts. N. Engl. J. Med..

[24] Vesely TM, Siegel JB (2005). Use of the peripheral cutting balloon to treat hemodialysis-related stenoses. J. Vasc. Interv. Radiol..

[25] Karnabatidis D (2013). Stent-grafts versus angioplasty and/or bare metal stents for failing arteriovenous grafts: A cross-over longitudinal study. J. Nephrol..

[26] Chen YY, Wu CK, Lin CH (2020). Outcomes of the Gore Excluder abdominal aortic aneurysm leg endoprosthesis for treatment of central vein stenosis or occlusion in patients with chronic hemodialysis. J Vasc. Surg. Venous Lymphat. Disord..

[27] Gong M, Zhou Y, Zhao B, Kong J, He X (2020). Efficacy of stent-graft placement to salvage central vein stents with recalcitrant restenosis in patients with arteriovenous fistulas. Semin. Dial..

[28] Vachharajani TJ, Taliercio JJ, Anvari E (2021). New devices and technologies for hemodialysis vascular access: A review. Am. J. Kidney Dis..

[29] Huang EP (2020). Undersized stent graft for treatment of cephalic arch stenosis in arteriovenous hemodialysis access. Sci. Rep..

