# Supplementary material for: Clinical outcomes and predictive factors of stent grafts treatment for symptomatic central venous obstruction in end stage kidney disease patients with arteriovenous access
Source: Sci Rep. 2024 Jun 3;14:12709. doi: 10.1038/s41598-024-63287-2 (PMC11148013; doi:10.1038/s41598-024-63287-2)
Supplement: Supplementary file 1 — Supplementary Tables. [file 41598_2024_63287_MOESM1_ESM.pdf]

Clinical outcomes and predictive factors of stent grafts treatment for symptomatic central venous obstruction in end stage kidney disease patients with arteriovenous access

Yamin Liu[1], Yufei Wang[1], Xinfang Wang[1], Beihao Zhang[1], Xiaoqing Lu[1], Xianhui Liang[1], Pei Wang[1]

[1]Blood Purification Center, Institute of Nephrology, the First Affiliated Hospital of Zhengzhou University, Zhengzhou, P. R. China

Corresponding Author: Pei Wang

Blood Purification Center, Institute of Nephrology, 1 East Jianshe Road

Zhengzhou, Henan Province, 450052, P. R. China. China

Tel: +86-0371-66295911

Mail: wpei@zzu. edu. cn

Supplement Table S1. The baseline characteristics of AVF/AVG

	AVF (N=47, Percent or Mean ± SD)	AVG (N=12, Percent or Mean ± SD)
Configuration		
Radial artery-Cephalic vein	44 (93.6)	0
Brachial artery-Perforating vein	1 (2.1)	0
Ulnar artery-Basilic vein	1 (2.1)	0
Radial artery-Perforating vein	1 (2.1)	0
Brachial artery-Basilic vein	0	8 (66.7)
Brachial artery-Cephalic vein	0	3 (25.0)
Brachial artery-Axillary Vein	0	1 (8.3)
Location		
Left upper extremity	27 (57.4)	11 (91.7)
Right upper extremity	20 (42.6)	1 (8.3)

Abbreviation: *AVF* arteriovenous fistula, *AVG* arteriovenous graft.

Supplement Table S2. Types of stent grafts (SGs)

	Viabahn™	Excluder™	Fluency™ Plus	Total
Subclavian vein	16	1	0	17
Brachiocephalic vein	22	11	1 (+1)	34 (+1)
Superior vena cava	5	3 (+1)	0	8 (+1)
Total	43	15 (+1)	1 (+1)	59 (+2)

Two patients placed 2 SGs during one procedure.
